# Therapeutic method for early-stage second primary non-small lung cancer: analysis of a population-based database

**DOI:** 10.1186/s12885-021-08399-y

**Published:** 2021-06-04

**Authors:** Congcong Chen, Zixiang Wu, Ziheng Wu, Chuanqiang Wu, Qi Wang, Tianwei Zhan, Lingjun Dong, Shuai Fang, Ming Wu

**Affiliations:** 1 The Second Affiliated Hospital, Zhejiang University School of Medicine, No. 88 Jiefang road, Hangzhou City, 310000 Zhejiang Province China; 2 School of Electrical and Information Engineering, AnHui University of Technology, Maanshan, China

**Keywords:** Second primary lung cancer, Type of surgery, Prognostic factors, Overall survival, Disease-free interval

## Abstract

**Background:**

Early-stage non-small lung cancer patients may survive long enough to develop second primary lung cancers. However, few studies have accurately described the therapeutic method, evaluation or prognostic factors for long-term survival in this complex clinical scenario.

**Methods:**

Patients who had first and second primary non-small lung cancer in the Surveillance, Epidemiology, and End Results database between 2004 and 2015 were evaluated. Patients were included when their tumors were pathologically diagnosed as non-small lung cancer and in the early-stage (less than 3 cm and with no lymph node metastasis). Therapeutic methods were categorized as lobectomy, sublobectomy or no surgery. The influence of different therapeutic methods on the overall survival rate was compared.

**Results:**

For the first primary tumor, patients who underwent lobectomy achieved superior survival benefits compared with patients who underwent sublobectomy. For the second primary tumor, long-term survival was similar in patients who underwent lobectomy and those who underwent sublobectomy treatment. The multivariate analysis indicated that age, disease-free time interval, sex, and first and second types of surgery were independent prognostic factors for long-term survival. Our results showed that the 5-year overall survival rate was 91.9% when the disease-free interval exceeded 24 months.

**Conclusion:**

Lobectomy for the first primary tumor followed by sublobectomy for the second primary tumor may be a beneficial therapeutic method for patients. If the disease-free interval exceeds 24 months, the second primary tumor will have no influence on the natural course for patients diagnosed with a first primary non-small lung cancer.

**Supplementary Information:**

The online version contains supplementary material available at 10.1186/s12885-021-08399-y.

## Introduction

Although non-small cell lung cancer (NSCLC) remains the leading cause of cancer-related death worldwide, the early detection rate has notably increased with the widespread use of diagnostic methods, such as high-resolution computed tomography. As a result, early-stage patients may survive long enough to develop multiple primary NSCLCs [1, 2]. Moreover, survivors of NSCLC have an approximately four to six times higher incidence of developing a second primary NSCLC than developing a first primary NSCLC [3, 4]. The International Association for the Study of Lung Cancer reports that the number of patients with multiple tumor nodules has increased since 2007 [5]. In our experience, thoracic surgeons are often required to evaluate which treatment is most beneficial and to identify prognostic factors for these patients.

However, few studies have accurately described the therapeutic method, evaluation or prognostic factors for long-term survival in this complex clinical scenario. Accordingly, there is no universal consensus regarding therapeutic treatment, and the 5-year overall survival (OS) rate for these patients ranges from 0 to 80% [6, 7]. The major weaknesses of related studies include a relatively small sample size and the fact that the second primary cancer was treated as a metastasis from the primary NSCLC.

In this study, we investigated which treatment is beneficial for early-stage first and second primary NSCLC using a large database.

## Methods

We selected patients from the latest version of the Surveillance, Epidemiology, and End Results (SEER) 18 database (1973 to 2015), a population-based cancer database that contains approximately 26% of the United States population [8]. Patients who were recorded at least twice in this database between 2004 and 2015 were evaluated. We excluded patients who were not pathologically diagnosed with lung cancer,who had other carcinomas, whose SEER stage was regional, and who received radiation or chemotherapy. When important information (such as age, sex, survival time and follow-up status) was unknown, the patient was excluded.

The major inclusion criteria in our study were that patients were pathologically diagnosed (code 1) with non-small lung cancer (small lung cancer were excluded) and that the tumors were in the early-stage (less than 3 cm and with no evidence for lymph node metastasis). To select patients who were diagnosed with a second primary NSCLC, the Martini and Melamed criteria were applied [9] as follows: (1) tumors with different histological types; and (2) tumors with similar histological types, if the disease-free interval (DFI) was more than 24 months or in different lobes and with no mediastinal lymph node metastasis (inclusion and exclusion criteria are in supplemental table).

The baseline demographics and characteristics of all patients (such as age at both diagnoses, sex, and race), characteristics of both primary tumors (such as histology, tumor size, tumor site and grade), and therapeutic method for both primary tumors were all collected from the SEER database. In this study, the therapeutic methods were categorized as lobectomy (code 33), sublobectomy (codes 20, 21 and 22) or no surgery (code 0), and other therapeutic methods were excluded (such as laser ablation or cryosurgery). Pathological types were divided into three types: adenocarcinoma, squamous cell carcinoma, and other pathological types (such as large cell carcinoma). The tumor site relationship for both tumors was classified as bilateral or ipsilateral. The DFI was defined as the recorded time interval between the first and second primary lung cancers. OS was defined as the time of diagnosis of the first primary lung cancer to either the date of death as a result of any cause or the last follow-up. This research was conducted ethically in accordance with the World Medical Association Declaration of Helsinki and was approved by the Ethics Committee of the Second Affiliated Hospital of Zhejiang University (ID: No. 2021–0486). The waiver for the informed consent was obtained from the Second Affiliated Hospital of Zhejiang University ethics committee given the retrospective nature of the study. A data use agreement was received form from the SEER administration.

### Statistical analysis

The Kaplan-Meier method was used for the calculation. Univariate Cox proportional hazards models were employed to analyze clinically interesting variables that may affect long-term survival, including continuous variables (age at first diagnosis, time interval until the second NSCLC, size of the first and second tumors) and categorical variables (sex, numbers of primary tumors, tumor site relationship, tumor histologic type, grade, and type of surgery for both tumors). Variables with a *p* value less than 0.1 in the univariate analysis were entered into the multivariate analysis, and variables with a *p* value less than 0.05 were considered to show a significant association with long-term survival. Hazard ratios (HRs) with 95% confidence intervals (CIs) are reported, and SPSS version 23.0 (IBM Corporation, Armonk, NY, USA) was utilized for all calculations.

### Cutoff point for the DFI

All possible cutoff points for the DFI were assessed using X-title version 3.6.1 (Yale University, https://medicine.yale.edu/lab/rimm/research/software.aspx) [10]. Patients were divided into two populations (“high” and “low” subsets), and differences in survival between these two populations were calculated using a standard Kaplan-Meier log-rank test. We chose the cutoff point at which the two groups had the highest chi-square value as the optimal cutoff point for the DFI between the first and second primary tumors.

### Deep learning and survival prediction

**Fig. 1:**
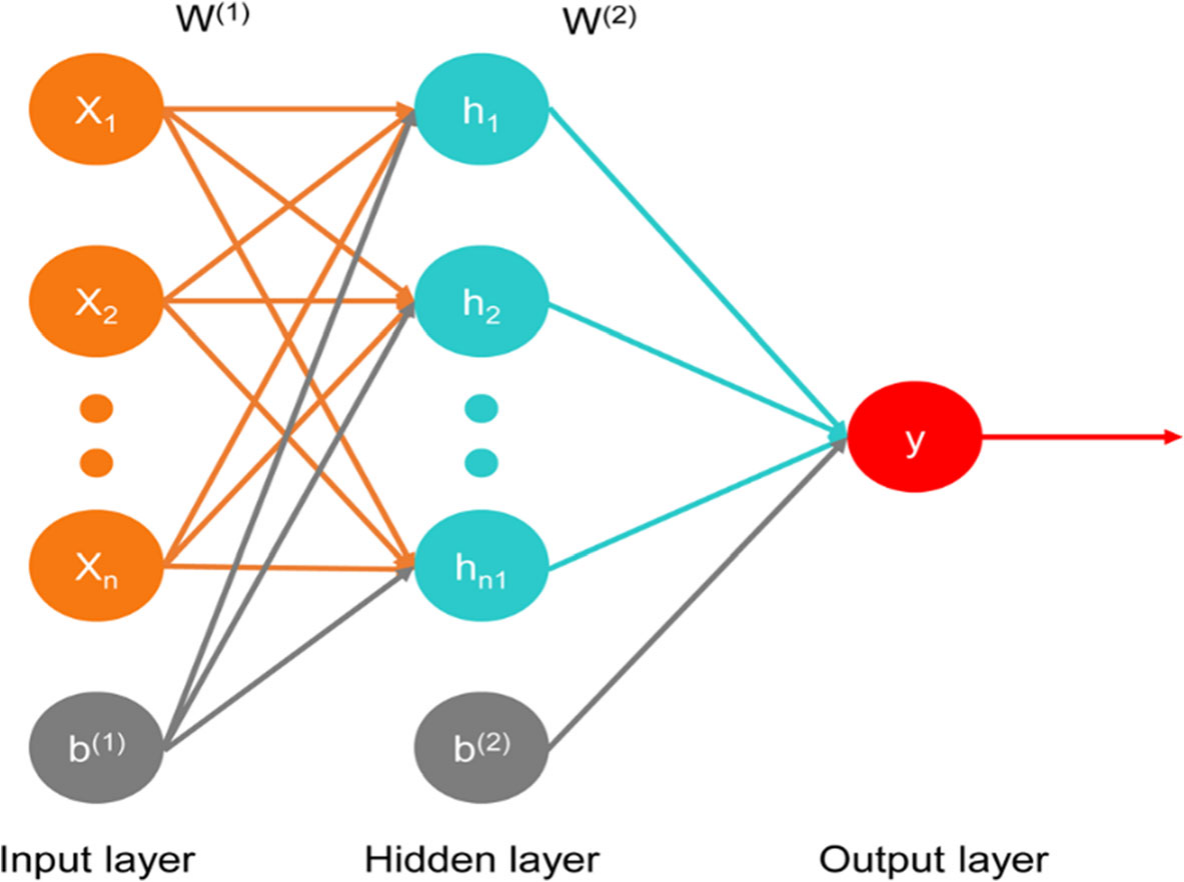
Topological structure of the back propagation neural network. Assuming that the network has *n* inputs and that *x* is the input vector, there are *n*_1_ neurons in the hidden layer. Here, *W* = {*w*^(1)^, *w*^(2)^} denotes the weights of both layers, *B* = {*b*^(1)^, *b*^(2)^} denotes the biases of both layers, and y is the output layer

In this study, we applied the back propagation (BP) neural network to construct a neural network model for predicting whether a patient would remain alive after a specific period of time (60 months). The topological structure of the BP neural network used in this paper is shown in Fig. 1; MATLAB version R2015a software was used (The MathWorks, Natick, MA, USA). First, the main factors that were independent prognostic factors for long-term survival in the multivariate analyses were normalized and then applied to train the BP neural network. In the output layer, 0 denotes “alive”, and 1 denotes “dead”. Second, the number of neurons in the hidden layer *n*_1_ was set to 50. The maximum number of iterations was set to 1000, and the learning rate *η* was set to 0.01. The convergence condition parameter *ξ* was set to 1E-4. The BP neural network algorithm converged if the difference in the loss function between two epochs of iteration was less than *ξ*. Finally, the first 200 samples were used to test the accuracy rate of the prediction model.

## Results

### Baseline demographics

From 2004 to 2015, a total of 1075 patients who were identified as having more than two early-stage NSCLC tumors were included in this study. Baseline demographics are shown in Tables 1 and 2. The mean age at diagnosis of the first primary lung cancer was 68.8 years, and the mean age at diagnosis of the second primary lung cancer was 70.6 years. The mean time interval between the two tumors was 21.8 months, and adenocarcinoma was the main histological type for both tumors (60.4% for the first and 53.6% for the second) (Table 1).

**Table 1:** Baseline demographics of patients with first and second primary lung cancer

	1st primary (*N* = 1075)	2nd primary (N = 1075)
Age (mean, y)	68.8 (9.0)	70.6 (9.0)
Histology		
Adenocarcinoma	649 (60.4%)	576 (53.6%)
Squamous cell	245 (22.8%)	196 (18.2%)
Other	181 (16.8%)	303 (28.2%)
Tumor size (mean, mm)	18.7 (6.4)	15.4 (6.3)
Tumor site		
Upper	680 (63.3%)	625 (58.1%)
Middle	50 (4.6%)	61 (5.7%)
Lower	332 (30.9%)	377 (35.1%)
Not determined	13 (1.2%)	12 (1.1%)
Grade		
Well	195 (18.1%)	213 (19.8%)
Moderately	402 (37.4%)	327 (30.4%)
Poorly or undifferentiated	301 (28.0%)	189 (17.6%)
Not determined	177 (16.5%)	346 (32.2%)
SEER stage		
In situ	5 (0.5%)	0 (0.0%)
Localized	1070 (99.5%)	1075 (100.0%)
–	–	–
Types of surgery		
Lobectomy	538 (50.1%)	236 (22.0%)
Sublobectomy	311 (28.9%)	395 (36.7%)
No surgery	226 (21.0%)	444 (41.3%)

**Table 2:** Baseline demographics and characteristics for all patients

	All patients (N = 1075)
Sex	
Female	675 (62.8%)
Male	400 (37.2%)
Marital status	
Married	566 (52.6%)
Single	462 (43.0%)
Unknown	47 (4.4%)
Race	
White	927 (86.2%)
Black	89 (8.3%)
Other	59 (5.5%)
Time interval (months)	21.8 (28.9)
Surgical sequence	
Surgery - Surgery	609 (56.7%)
Surgery - No surgery	240 (22.3%)
No surgery - Surgery	22 (2.0%)
No surgery - No surgery	204 (19.0%)
Tumor site relation	
Bilateral	698 (64.9%)
Ipsilateral	377 (35.1%)

Greater than half (609 of 1076, 56.7%) of the patients underwent surgery for both lung cancers, whereas 362 patients (24.3%) underwent surgery for only one cancer (Table 2). Nonetheless, the majority of patients (79%) underwent an operation forfirst primary lung cancer. For the second primary lung cancer, approximately half of the patients (41.3%) did not undergo surgery, and only 22.0% underwent lobectomy (Table 1).

### Prognostic factors and OS

The results of the univariate and multivariate analyses of prognostic factors related to OS are shown in Table 3. In the univariate analysis, three or more primary lung cancers were not significantly related to the patients’ long-term survival outcomes. The factors significantly related to good long-term outcomes included a young age, long time interval, small tumor size for both tumors, female sex, ipsilateral tumor site relationship, both adenocarcinomas, both with well and moderate differentiation, and surgical treatment for both tumors. Moreover, the multivariate analysis indicated that age (*p* < 0.001), time interval (*p* < 0.001), sex (*p* = 0.001), first type of surgery (*p* = 0.034) and second type of surgery (*p* = 0.004) were independent prognostic factors for long-term survival.

**Table 3:** Univariate andmultivariate analysis of overall survival predictors for patients with multiple primary lung cancers

Characteristics	Univariate Analysis		Multivariate Analysis	
	HR (95% CI)	*p* Value	HR (95% CI)	*p* Value
**Continuous variables**				
Age (y)	1.045 (1.033–1.058)	< 0.001	1.042 (1.024–1.060)	< 0.001
Time interval (months)	0.976 (0.972–0.980)	< 0.001	0.973 (0.967–0.979)	< 0.001
First tumor size (mm)	1.020 (1.005–1.036)	0.008	1.010 (0.988–1.048)	0.377
Second tumor size (mm)	1.024 (1.008–1.040)	0.003	1.023 (0.999–1.048)	0.064
**Categorical variables**				
Sex		0.016		0.001
Female	0.782 (0.641–0.956)		0.634 (0.479–0.838)	
Male	Ref. level		Ref. level	
Number of primary		0.482		
Two	1.126 (0.809–1.568)			
Three or more	Ref. level			
Tumor site relationship		0.035		0.663
Ipsilateral	1.256 (1.016–1.551)		1.064 (0.805–1.406)	
Bilateral	Ref. level		Ref. level	
Frist histology		0.003		0.233
AC	0.680 (0.524–0.883)	0.004	0.719 (0.492–1.051)	0.089
SCC	0.933 (0.696–1.252)	0.645	0.807 (0.523–1.243)	0.330
Others	Ref. level		Ref. level	
Second histology		0.008		0.332
AC	0.701 (0.559–0.878)	0.002	0.887 (0.611–1.286)	0.526
SCC	0.857 (0.646–1.136)	0.282	0.723 (0.464–1.125)	0.150
Others	Ref. level		Ref. level	
Frist grade		0.048		0.285
Well and moderately	0.799 (0.639–0.998)		0.847 (0.624–1.149)	
Poorly and undifferentiated	Ref. level		Ref. level	
Second grade		0.004		0.256
Well and moderately	0.678 (0.522–0.882)		0.835 (0.613–1.139)	
Poorly and undifferentiated	Ref. level		Ref. level	
Frist types of surgery		< 0.001		0.034
Lobectomy	0.278 (0.216–0.357)	< 0.001	0.570 (0.328–0.992)	0.047
Sublobectomy	0.478 (0.367–0.623)	< 0.001	0.805 (0.461–1.407)	0.447
No surgery	Ref. level		Ref. level	
Second types of surgery		0.009		0.004
Lobectomy	0.707 (0.538–0.929)	0.013	0.454 (0.282–0.731)	0.001
Sublobectomy	0.749 (0.601–0.934)	0.010	0.549 (0.363–0.829)	0.004
No surgery	Ref. level		Ref. level	

*AC* adenocarcinoma, *SCC* squamous cell carcinoma

**Fig. 2:**
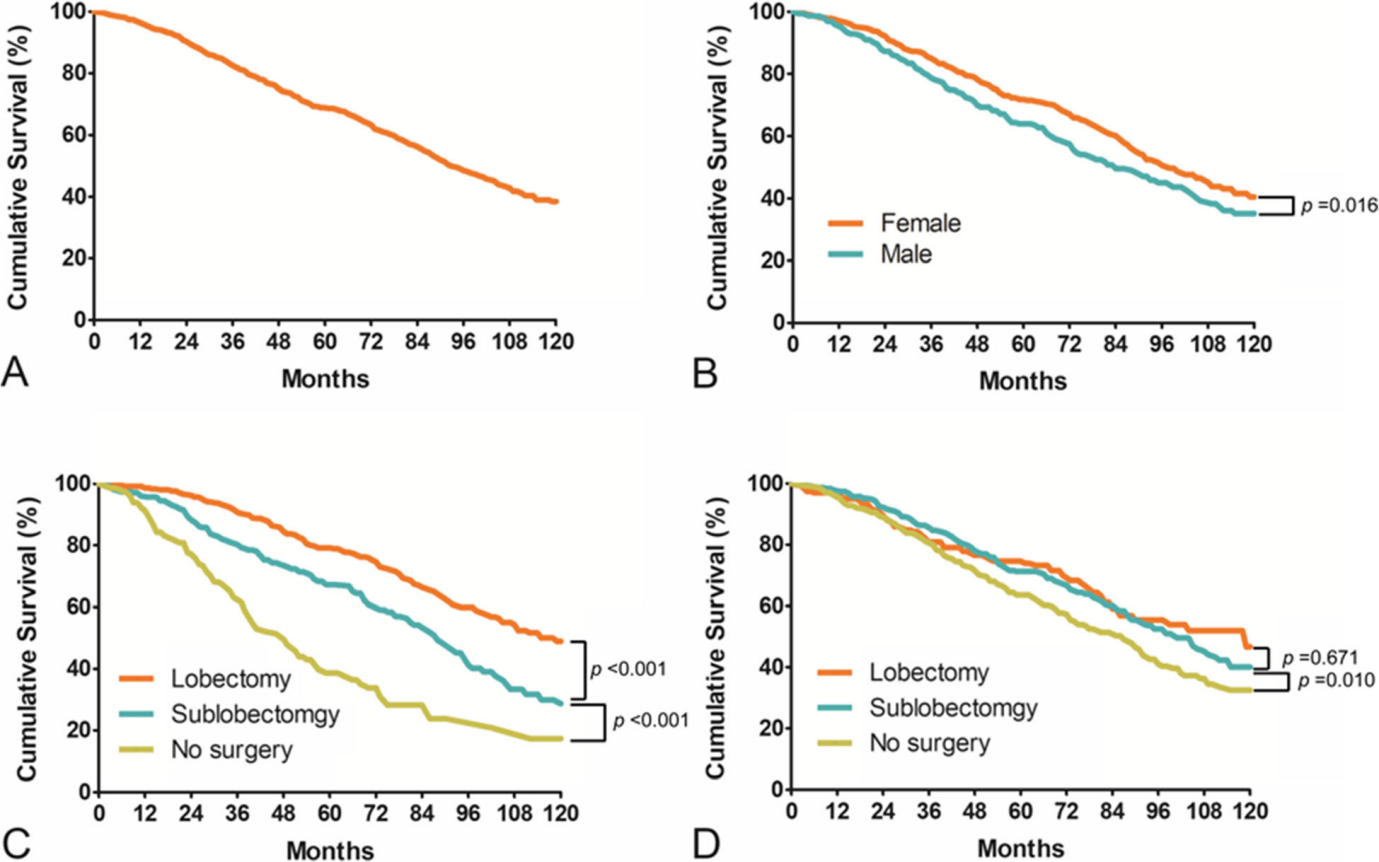
**A**: Overall survival rate for all patients; the 5-year overall survival rate was 68.8%. **B**: The 5-year OS rate for males was significantly lower than that for females (*p* < 0.001). **C**: For the first primary tumor, patients who underwent lobectomy achieved superior survival benefits than patients who underwent sublobectomy (*p* < 0.001). **D**: For the second primary tumor, patients who underwent lobectomy or sublobectomy had similar 5-year survival rates (*p* = 0.671)

For all patients, the median OS time was 92 months. The 3-year OS rate was 82.5%, with 5-year and 10-year OS rates of 68.8 and 38.4%, respectively (Fig. 2A). The survival analysis based on the log-rank test indicated that the 5-year OS rate for females was 71.9%, which was significantly better than the 5-year OS rate for males (64.0%; HR, 0.782; 95% CI, 0.641–0.956; *p* = 0.016) (Fig. 2B).

### Treatment choice

For the first primary tumor, patients who underwent lobectomy and sublobectomy had 5-year survival rates of 79.2 and 67.3%, respectively (*p* < 0.001) (Fig. 2C). The results also showed that patients who underwent lobectomy achieved superior survival outcomes than patients who underwent sublobectomy (HR, 0.576; 95% CI, 0.456 to 0.727; *p* < 0.001). For the second primary tumor, patients who underwent lobectomy (74.6%) or sublobectomy (71.2%) had similar 5-year survival rates (HR, 1.063; 95% CI, 0.800 to 1.413; *p* = 0.671) (Fig. 2D).

**Fig. 3:**
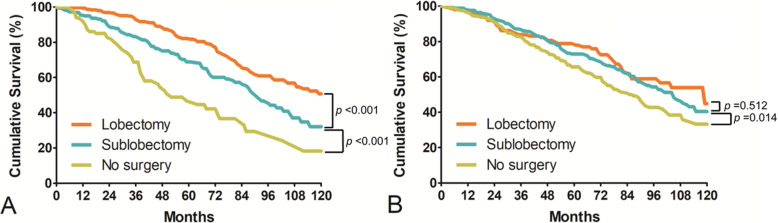
**A**: For patients with a first primary tumor less than 2 cm, the overall survival rates for those who received lobectomy or sublobectomy were significantly different (*p* < 0.001). **B**: For patients with a second primary tumor less than 2 cm, the overall survival rates for patients who underwent lobectomy or sublobectomy were not significantly different (*p* = 0.512)

We also investigated the survival rate according to the subclassification of tumor size, which was less than 2 cm. Lobectomy (82.2%) was associated with superior survival outcomes compared with sublobectomy (68.9%) for the first primary tumor (*p* < 0.001) (Fig. 3A). In contrast, no significant differences in the long-term survival outcomes were noted between lobectomy (78.9%) and sublobectomy (72.9%) for the second primary tumor (*p* = 0.512) (Fig. 3B).

### Optimal cutoff point

**Fig. 4:**
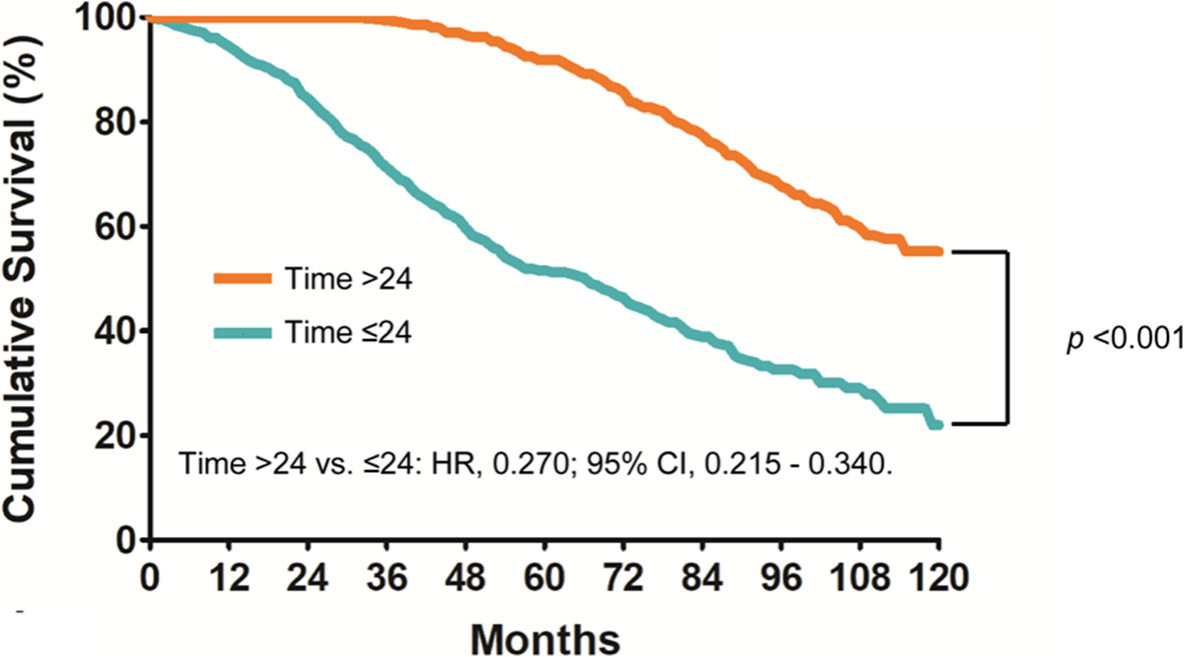
The optimal cutoff point for the disease-free interval was 24 months, and all-cause mortality was significantly reduced when the disease-free interval exceeded 24 months (5-year OS, 91.9% vs. 51.6%, *p* < 0.001)

All of the possible cutoff points for the DFI between the first and second primary tumors were assessed. Because survival is of great importance to patients, we selected the maximum difference in survival as the cutoff point for the time interval (24 months). The survival analysis showed that after adjusting for other prognostic factors (including sex, age, size of the second tumor and therapeutic treatment for both tumors), all-cause mortality was significantly reduced when the DFI exceeded 24 months (5-year OS, 91.9% vs. 51.6%. HR, 0.270; 95% CI, 0.215–0.340; *p* < 0.001) (Fig. 4).

All of the independent prognostic factors were applied to train the BP neural network for predicting patient survival at 60 months. The value of the cost function decreased with increasing iteration epoch, and the algorithm converged after 169 iteration epochs (supplemental Figure). After using the first 200 samples as the test dataset, the accuracy was 98%.

## Discussion

Multiple primary NSCLCs were initially described by Beyreuther [11] in 1924. Although almost one century has passed, the optimal treatment, cutoff point for the DFI and prognostic factors of this population remain unclear. Although Hamaji et al. [1] did not find any association between the DFI and long-term survival, several studies have indicated that a longer DFI might have a positive correlation with improved long-term survival [12]. Our results showed that the 5-year OS rate was 91.9% when the DFI exceeded 24 months. This OS rate was comparable to that of stage IA patients with single NSCLC according to the 8th edition of the American Joint Committee on Cancer’s TNM staging system (the 5-year OS rate for stage IA NSCLC is 90%) [13]. Therefore, it is hypothesized that if the DFI exceeds 24 months, the second primary tumor will have no influence on the natural course for patients diagnosed with a first primary NSCLC.

Although exploring the DFI between first and second tumors is important, it is more crucial to evaluate which therapeutic method is associated with prolonged survival times, given that patients are most concerned about this aspect. Our research showed that lobectomy results in improved long-term survival for patients with first primary NSCLC compared with sublobar resection. Over the past two decades, lobectomy has been considered the standard surgical procedure for the treatment of stage I primary NSCLC (tumor size less than 3 cm). However, for tumors less than 2 cm in size, numerous studies have discussed whether sublobar resection might provide an oncologic outcome similar to that of lobectomy [14, 15]. Using the SEER database, Dai et al. [14] recently reported that lobectomy was associated with superior survival compared with sublobar resection for patients with tumor sizes less than 2 cm. In addition, Zhang et al. [15] found that lobectomy led to prolonged OS for patients with tumors less than 2 cm in size, which is similar to our findings. Therefore, we conclude that lobectomy is the first choice for patients with primary NSCLC.

The majority of thoracic surgeons recommend surgical resection as the most valuable treatment for patients with a second primary lung cancer who can tolerate surgery [1, 16]. Nonetheless, the extent of resection remains an open issue because surgeons have not reached an agreement. Some studies suggest that another lobectomy should be the first choice [17], whereas other studies conclude that sublobar resection is acceptable [16]. Many factors influence the choice of the therapeutic method, including the first type of surgery as well as patient age and pulmonary function. In particular, the mean age at diagnosis of the second tumor among patients who underwent lobectomy for the first primary tumor was approximately 71 years, and it is important to balance the risks and benefits of a second lobectomy. For example, Mery et al. [18] demonstrated that the difference in long-term survival between patients who undergo lobectomy and limited resection would be negligible for patients older than 71 years. Other studies found that pulmonary function was significantly better when applying sublobar resection [19, 20]. In our study, lobectomy was not associated with any superiority in long-term survival compared with sublobar resection for second primary NSCLC. Indeed, the 5-year OS rate was 74.6% for lobectomy and 71.2% for sublobar resection (*p* = 0.671). Thus, it may be summarized that limited resection is an acceptable therapy with satisfactory long-term survival for a second NSCLC.

The multivariate analysis revealed that in addition to the time interval and type of surgery, sex and age were also associated with survival; other factors, including the tumor site relationship, histological type and more than three tumors, exhibited no relationship with survival. These results were consistent with other studies that clarified only one or two factors using smaller case series. For example, Finley et al. [21] demonstrated that female sex was an independent factor for improved survival and that the survival outcome was independent of the tumor site location. Moreover, Jiang et al. [22] indicated that survival might not correlate with histological type, and Zhang et al. [23] showed that more than three tumors would not affect long-term survival.

In our study, a BP neural network was applied as a deep learning (one of artificial intelligence) [24] method to assess these factors. The BP neural network can theoretically approximate any nonlinear continuous function under the conditions of a reasonable structure and appropriate weights. We used the first 200 samples as the test dataset and found that the accuracy of the BP neural network was approximately 98%. We further verified these independent prognostic factors through the test. When there is a lack of prospective and randomized studies on a given population, the use of a large sample size and deep learning can improve the quality of the evidence, which will provide valuable suggestions for surgeons to manage patients.

There are three limitations to this study. The first and major limitation is that this was a retrospective study. Although a large sample size and deep learning can provide relatively high-quality evidence, the SEER database does not record the criteria (for example: comorbidities and preserved pulmonary function) used for the selection of patients for surgery or for choosing the surgical strategy (or example: minimally invasive or open approach); therefore, selection bias cannot be eliminated. Second, lymph node permeation is the major origin of relapse for patients who receive sublobectomy in clinical early-stage NSCLC [25]. However, it is difficult to take this factor into consideration preoperatively to determine the indication for lobectomy or sublobectomy in a retrospective study. The authors believe that intraoperative pathologic N1 node assessment should be performed in patients who underwent sublobectomy. If positive, the surgical procedure was converted to lobectomy. Third, the detection of ground-glass opacity (GGO)-dominant adenocarcinoma has increased in recent years, and it is believed that patients with GGO-dominant tumors will have good long-term survival outcomes. Some studies have suggested that for tumor sizes less than 2 cm and GGO dominant (exceeding 50%) adenocarcinoma, sublobar resection may provide outcomes similar to those of lobectomy [26, 27], but further studies are needed to verify this conclusion. However, the rate of GGO is not reported in the SEER database, and we hope that these data will be provided in the future.

## Conclusion

In conclusion, lobectomy for the first primary tumor followed by sublobectomy for the second primary tumor may represent a beneficial therapeutic approach. If the DFI exceeds 24 months, the second primary tumor will have no influence on the natural course for patients diagnosed with a first primary NSCLC. In the first 24 months, close and careful follow-up is important for patients who have primary NSCLC.

## Supplementary Information

**Additional file 1: Table S1.** Inclusion and exclusion criteria in this study.

**Additional file 2.**

## Data Availability

The datasets used and analysed during the current study are available from http://www.seer. cancer.gov.
