# Supplementary material for: Therapeutic method for early-stage second primary non-small lung cancer: analysis of a population-based database
Source: BMC Cancer. 2021 Jun 4;21:666. doi: 10.1186/s12885-021-08399-y (PMC8176724; doi:10.1186/s12885-021-08399-y)
Supplement: Supplementary file 1 — Additional file 1: Table S1. Inclusion and exclusion criteria in this study. [file 12885_2021_8399_MOESM1_ESM.docx]

Supplemental Table: inclusion and exclusion criteria in this study

| Inclusion criteria | Exclusion criteria |
| --- | --- |
| (1). recorded at least twice between 2004 and 2015;  (2). pathologically diagnosed as non-small lung cancer;  (3). tumor size less than 3 cm;  (4). with no evidence for lymph node metastasis;  (5). meet Martini and Melamed criteria. | (1). SEER stage is regional;  (2). received radiation;  (3). received chemotherapy;  (6). received other therapeutic methods (such as laser ablation or cryosurgery)  (5). important information (such as age, sex, survival time and follow-up status) was unknown;  (6). had other carcinomas. |
