# Supplementary figures and images for: Therapeutic method for early-stage second primary non-small lung cancer: analysis of a population-based database
Source: BMC Cancer. 2021 Jun 4;21:666. doi: 10.1186/s12885-021-08399-y (PMC8176724; doi:10.1186/s12885-021-08399-y)

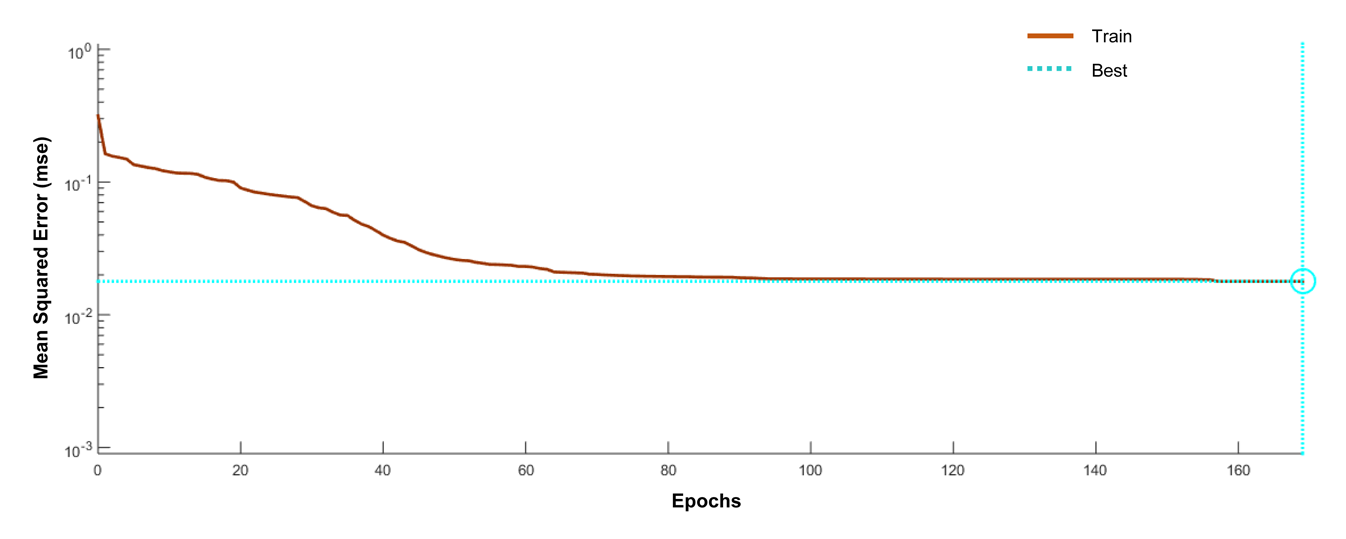

Supplement: Supplementary file 2 — Additional file 2. [file 12885_2021_8399_MOESM2_ESM.tif]
